# Supplementary material for: Inhibition of the mevalonate pathway enhances cancer cell oncolysis mediated by M1 virus
Source: Nat Commun. 2018 Apr 18;9:1524. doi: 10.1038/s41467-018-03913-6 (PMC5906622; doi:10.1038/s41467-018-03913-6)
Supplement: Supplementary file 1 — Supplementary Information [file 41467_2018_3913_MOESM1_ESM.pdf]

Inhibition of the Mevalonate pathway enhances cancer cell oncolysis mediated by M1 Virus

Liang et al.

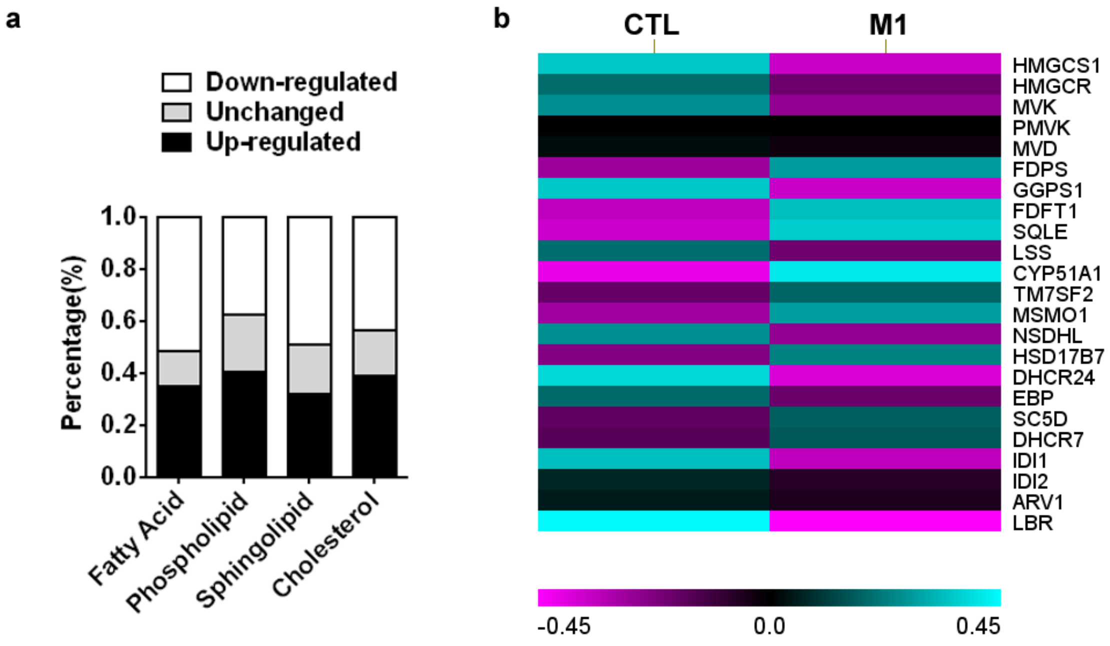

Supplementary Figure 1. Mevalonate pathway gene expression after M1 virus infection of Hep3B cells. Hep3B cells were treated with M1 virus (MOI=1) or vehicle. Twenty-four hours later, RNA was collected and analyze by GeneChip Human Genome U133 Plus 2.0 Array (Affymetrix). Microarray analysis was performed on three biological sample. (a) Analyzation of four kinds of lipid-related gene expression changes according to microarray data. (b) Heat maps of the mevalonate-cholesterol pathway related genes expression after M1 virus infection according to microarray data above.

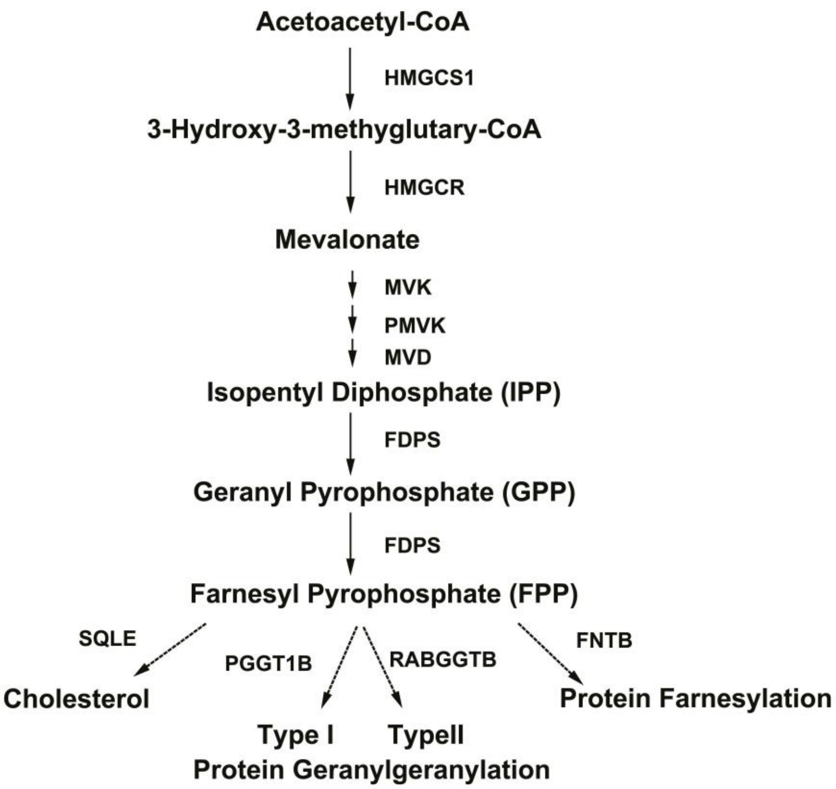

Supplementary Figure 2. Schematic of cholesterol biosynthesis pathway.

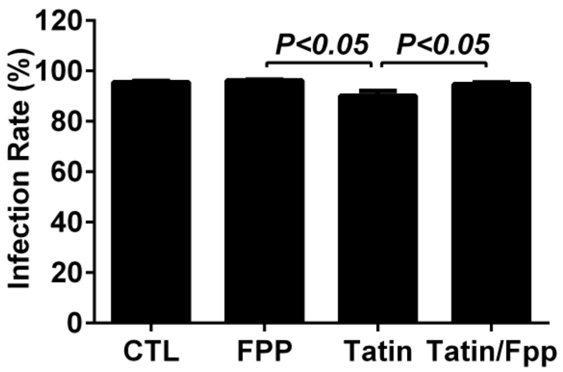

Supplementary Figure 3. HMG-CoA reductase inhibition did not enhance M1 virus infection in Hep3B cells. Hep3B cells were treated with Fluvastatin (5 μM), and FPP was added into the cells simultaneously at concentration of 30μM. Then cells were infected with M1-GFP virus (MOI=0.1) for 24 hours. M1 virus infection rate was detected by flow cytometry assay. n=3. Data shown was the mean ± s.e.m. Statistical significance was using one-way ANOVA.

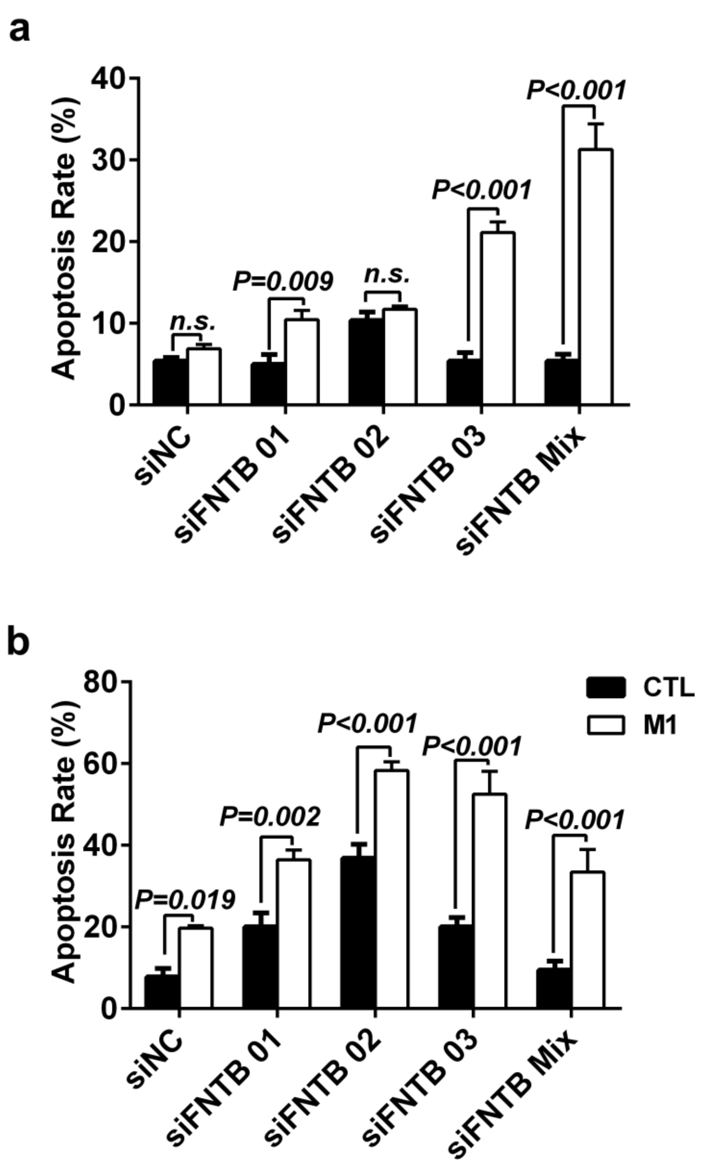

Supplementary Figure 4. M1 virus-induced cancer cell apoptosis rates were enhanced after FNTB knocking-down. HCT-116 (a) and SW1990 (b) cells were treated with negative control (NC) or FNTB siRNA for 24 hours, and infected with M1 virus (MOI=1). Cell apoptosis was determined with flow cytometry assay by annexin IV/ PI double-staining 48 hours after M1 virus infection. n=3. Data shown were the mean ± s.e.m. Statistical significance was using one-way ANOVA.

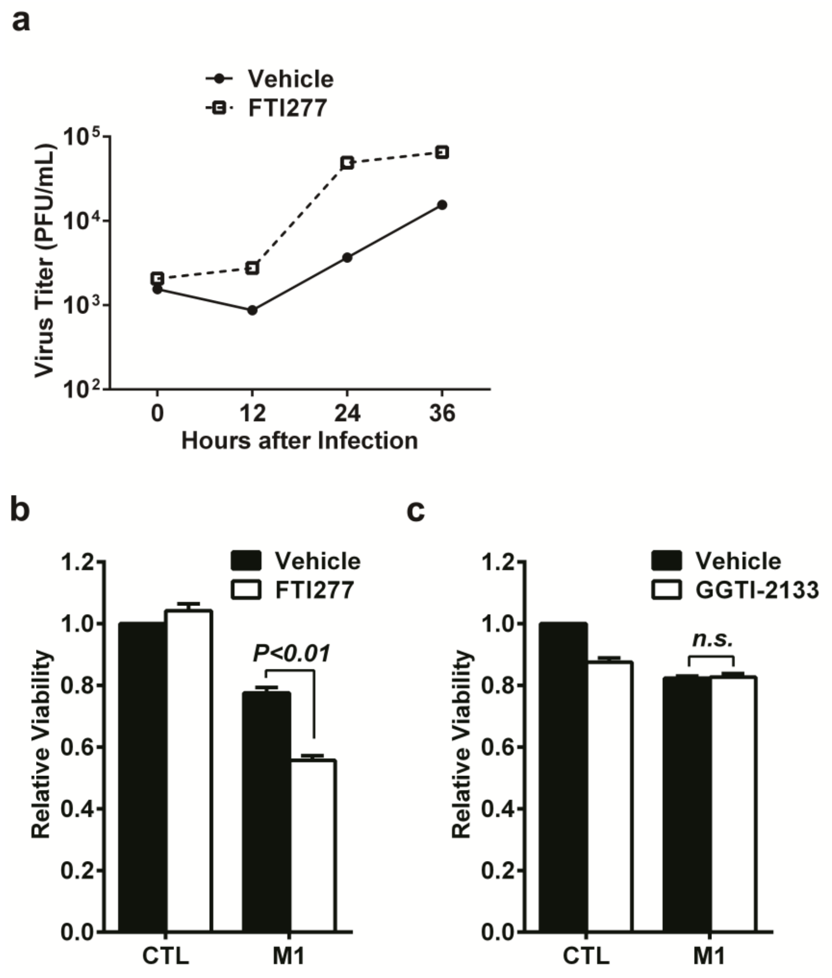

Supplementary Figure 5. Protein farnesylation rather than geranylgeranylation involved in M1 virus-induced cell death. (a) M1 virus titer was measured by TCID50 assay after FTI277 (4 μM) or vehicle treatment, and M1 virus infection (MOI=1) for 36 hours in HCT-116 cells. n=1. (b-c) HCT-116 cells were treated with vehicle, GGTI-2133 (5 μM) or FTI277 (4 μM), and infected with M1 virus (MOI=1). Cell viability was determined with MTT assay 72 hours after M1 virus infection. n=3. Data shown in b-c were the mean ± s.e.m. Statistical significance was using one-way ANOVA.

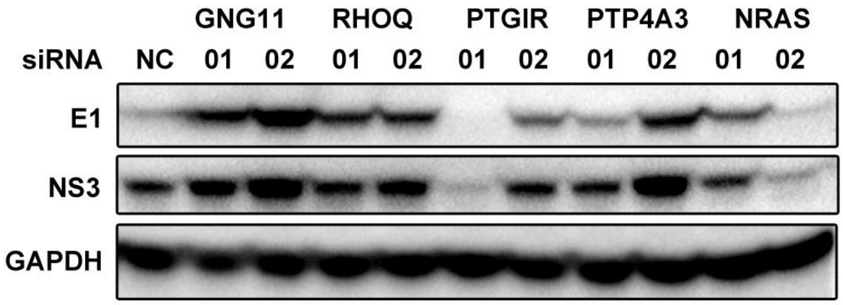

Supplementary Figure 6. Screening of five candidates by detecting virus proteins. HCT-116 cells were treated with negative control (NC) or siRNAs for 24 hours, and then infected with M1 virus (MOI=1). Cell lysates are collected 24 hours after infection, and western blotting assay was used to detect virus proteins. n=2.

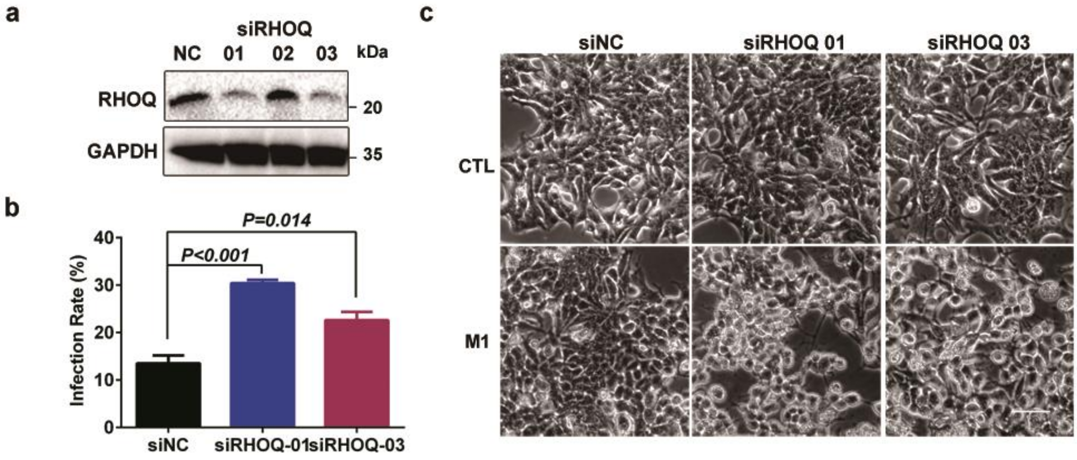

Supplementary Figure 7. The antiviral function of RHOQ was proved in SW1990 cells. (a) RHOQ protein was detected by western blot after NC or RHOQ siRNAs pretreatment for 48 hours in SW1990. n=2. (b) SW1990 cells were treated with negative control (NC) or siRNAs for 24 hours, and then infected with M1-GFP virus (MOI=1) for 24 hours. M1 virus infection rate was detected by flow cytometry assay. n=3. Data shown were the mean ± s.e.m. Statistical significance was using one-way ANOVA. (c) Phase contrast microscope images of SW1990 cells after NC or RHOQ siRNAs pretreatment for 24 hours and M1 virus infection (MOI=1) for another 48 hours. n=2. Scale bar: 100 μm.

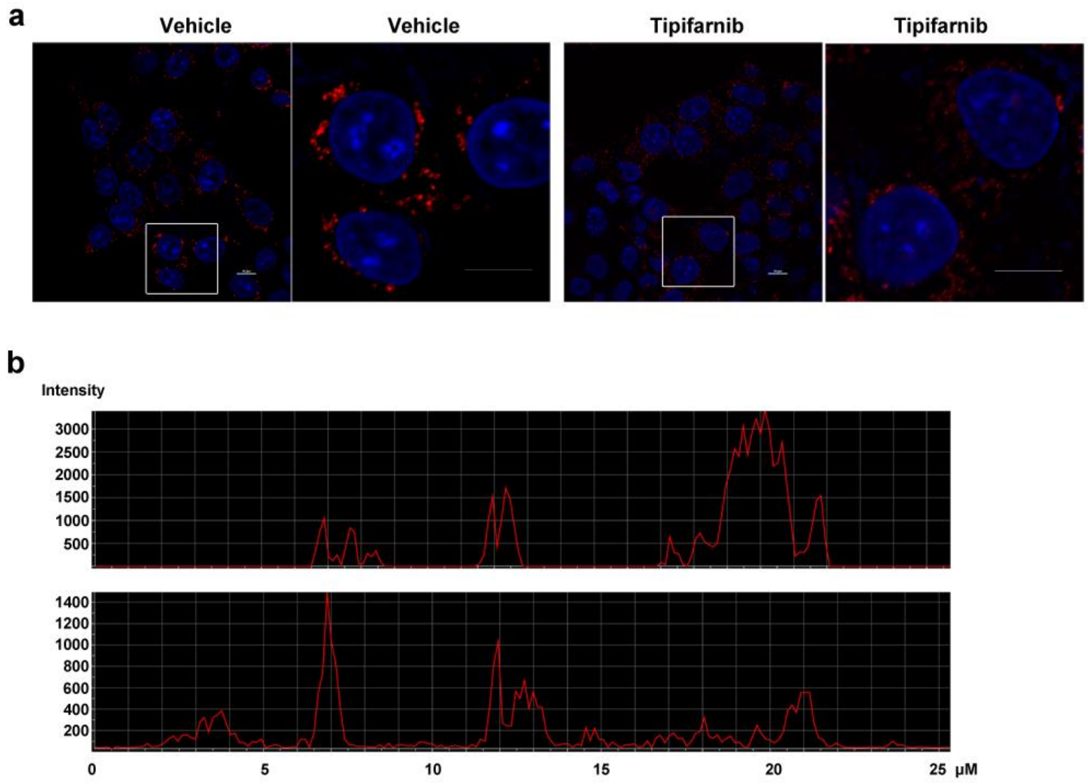

Supplementary Figure 8. RHOQ was dispersed by FTI. (a) RHOQ intracellular location study by laser scanning confocal microscope with or without Tipifarnib (50 nM) in HCT-116 cells. n=2. Scale bar: 10 μm. (b) Quantitative analysis of cell RHOQ expression in above figures by Nikon confocal software.

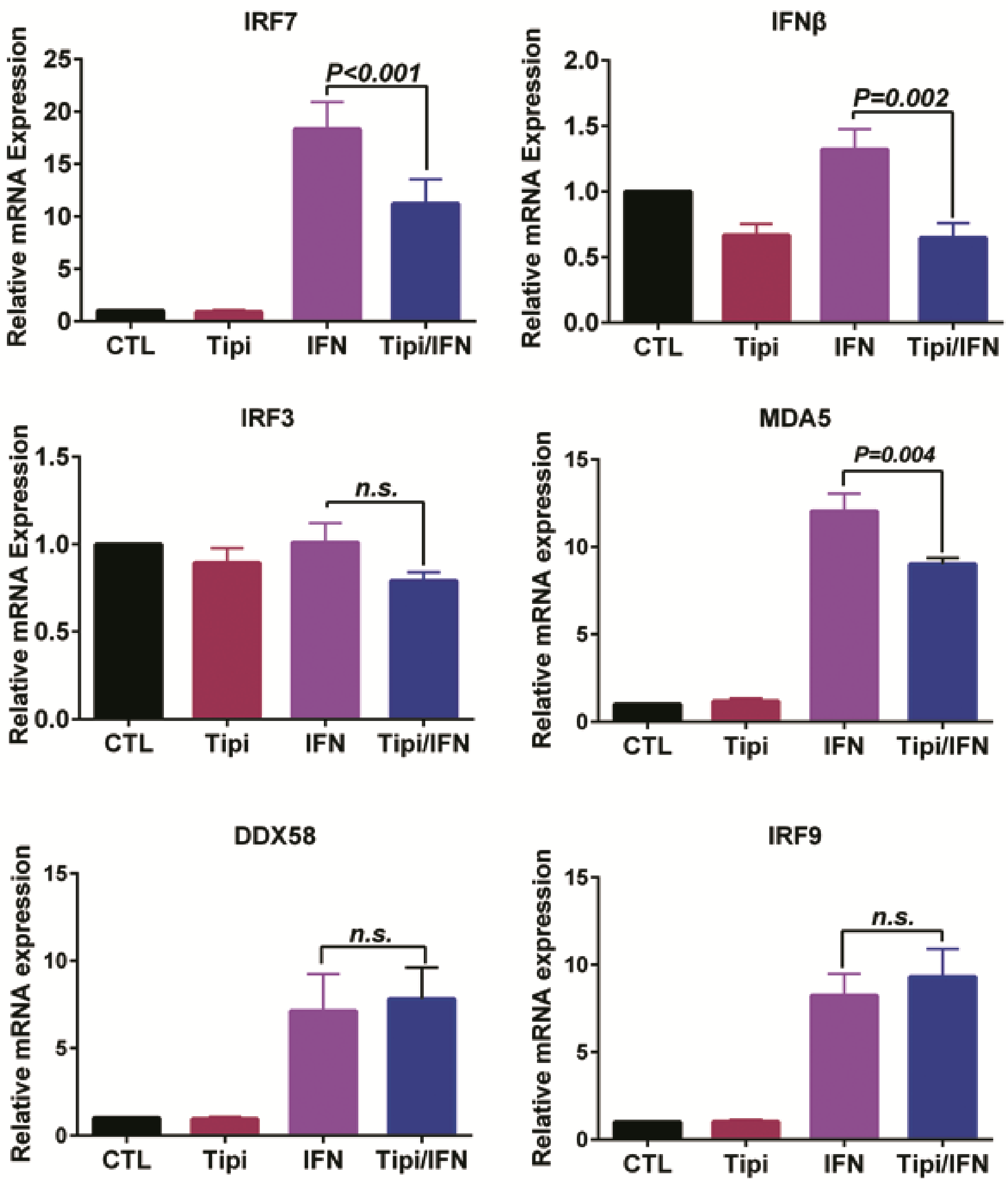

Supplementary Figure 9. Tipifarnib inhibited IFNβ-induced antiviral factor expressions. HCT-116 cells were pretreated with vehicle and Tipifarnib (50nM) for 6 hours, and treated with IFNβ（25ng/mL）. 12 hours later, RNA was harvested for qRT-PCR detection of interferon-stimulated gene expressions. n=3. Data shown were the mean ± s.e.m. Statistical significance was using one-way ANOVA.

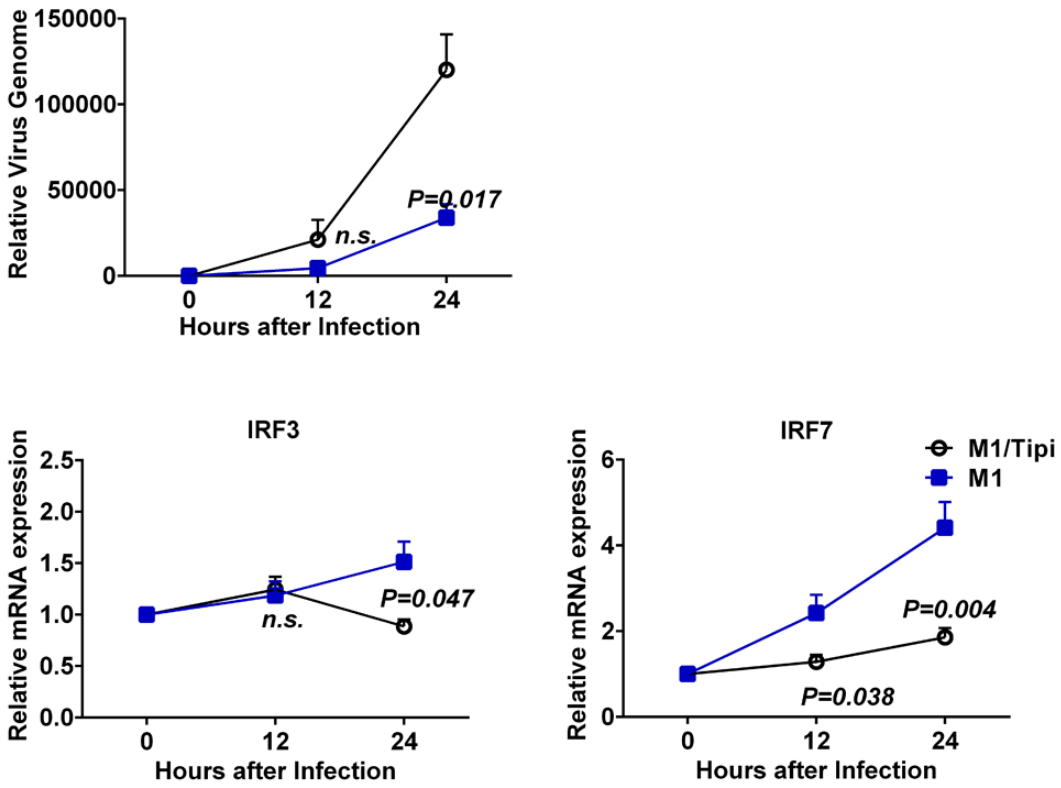

Supplementary Figure 10. Tipifarnib inhibited M1 virus-induced IRF3 and IRF7 expressions. HCT-116 cells were mock-infected or infected with M1 virus (MOI=1). 12 and 24 hours later, RNA was harvested for qRT-PCR detection of M1 virus gene and interferon-stimulated genes expression. n=3. Data shown were the mean ± s.e.m. Statistical significance was using t-test, two sided.

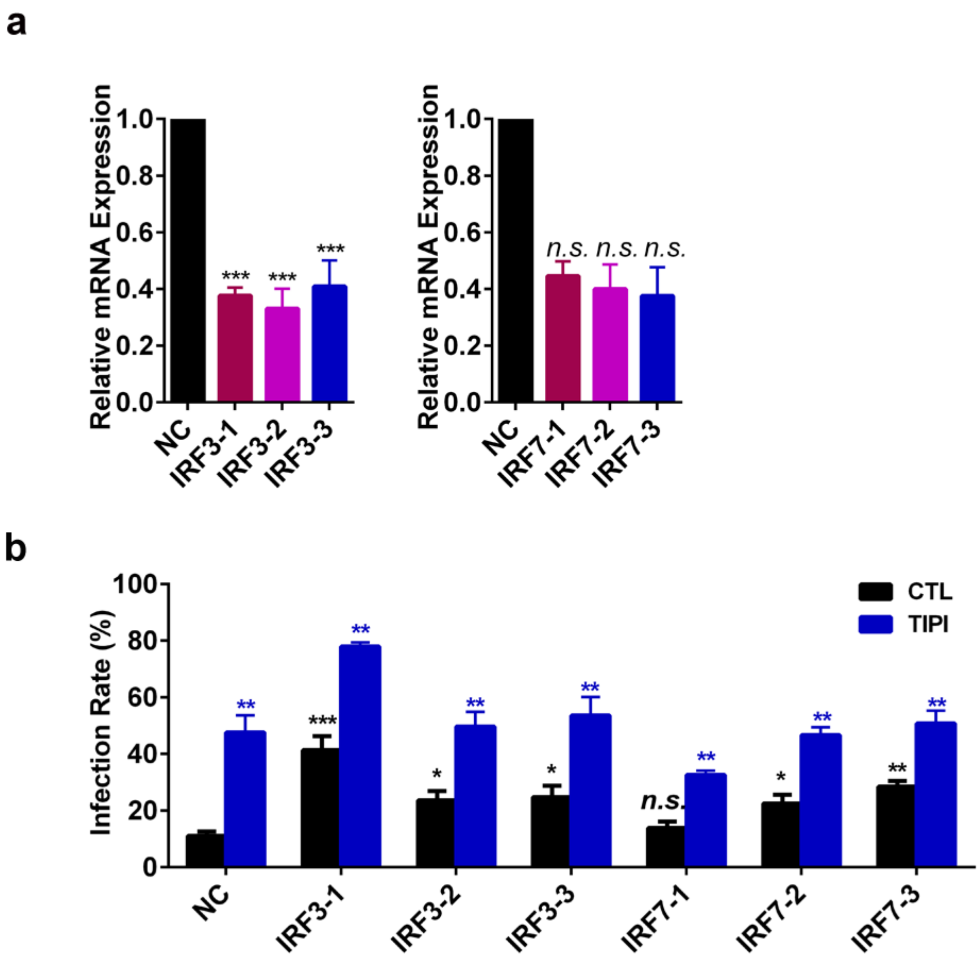

Supplementary Figure 11. Knock-down of IRF3 and IRF7 enhanced M1 virus infection. (a) Knock-down efficiency of siRNA. (b) HCT-116 cells were pretreated with NC, IRF3 or IRF7 siRNAs for 24 hours and infected with M1-GFP virus (1 MOI) or M1/ Tipi (50 nM) for another 48 hours. M1 virus infection rate was detected by flow cytometry assay. n=3. Data shown were the mean ± s.e.m. Statistical significance was using one-way ANOVA.

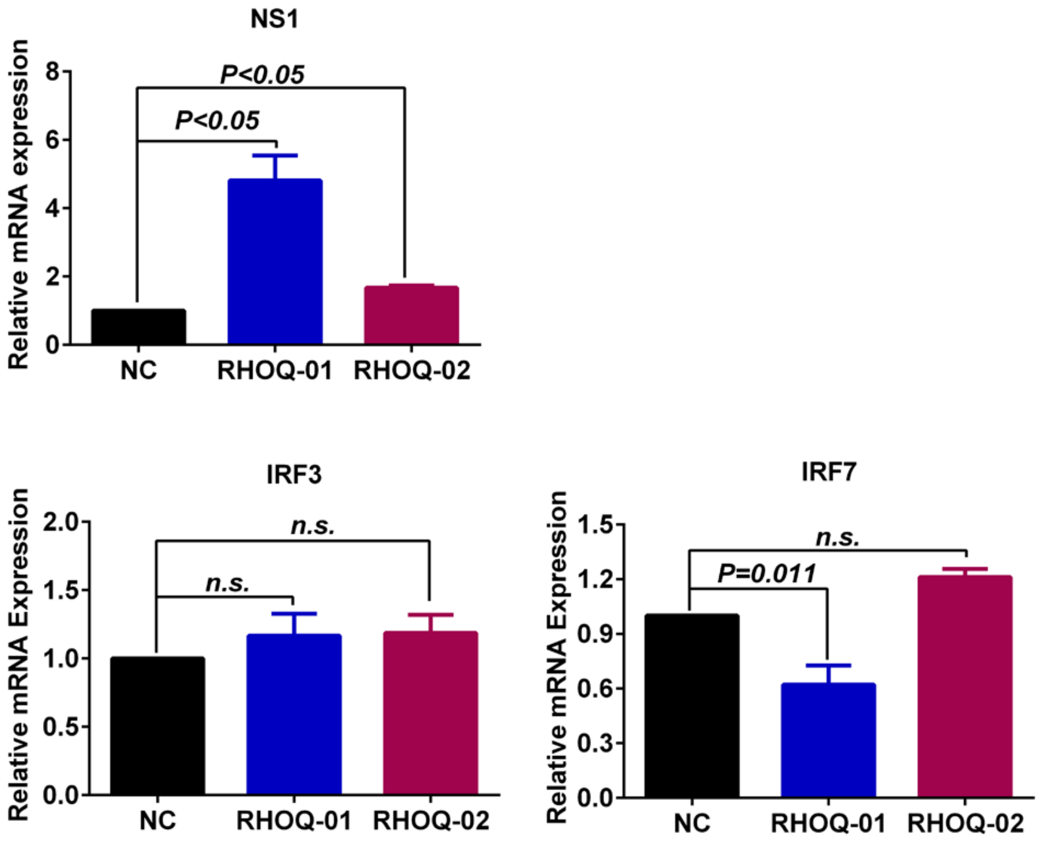

Supplementary Figure 12. Knocking-down of RHOQ did not suppress IRF3 and IRF7 mRNA expression. HCT-116 cells were treated with negative control (NC) or siRNAs for 48 hours, and then infected with M1 virus (MOI=1) for 24 hours. RNA was harvested for qRT-PCR detection of virus gene, IRF3 and IRF7 mRNA expressions. n=3. Data shown were the mean ± s.e.m. Statistical significance was using one-way ANOVA.

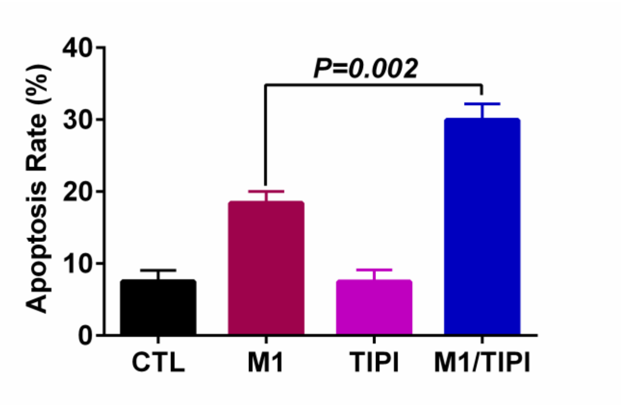

Supplementary Figure 13. Tipifarnib promoted M1 virus-induced cancer cell apoptosis. HCT-116 cells were treated with vehicle, Tipifarnib (50nM), M1 (MOI=1) or Tipifarnib /M1 for 48 hours. Flow cytometry detection of Annexin V/PI staining assay was used to detect cell apoptosis. n=3. Data shown were the mean ± s.e.m. Statistical significance was using one-way ANOVA.

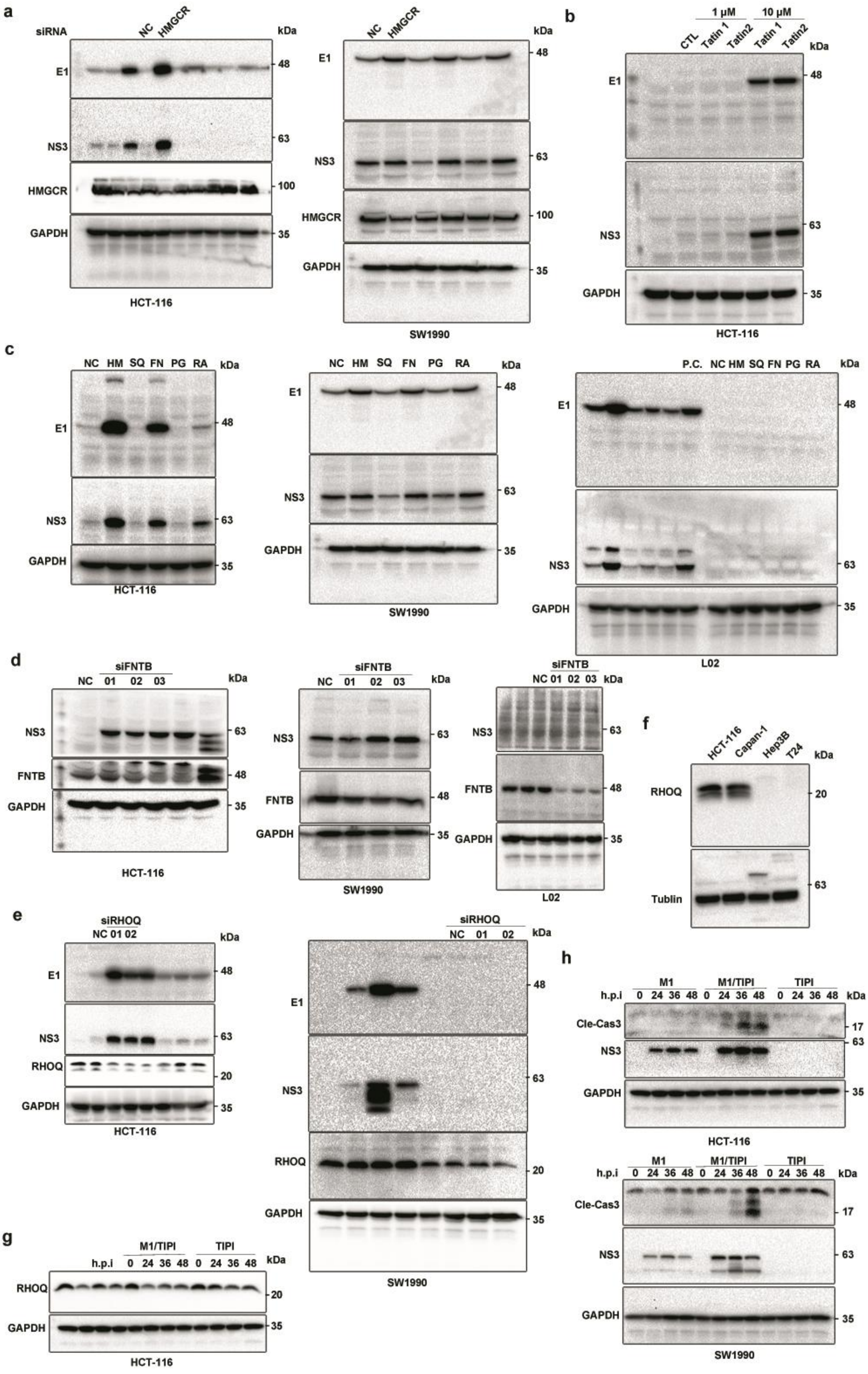

Supplementary Figure 14. Full western blots. Blots for Figures 2 d(a), 2e (b), 2g (c), 2h (d), 3e (e), 3g (f), 3i (g) and 5e (h).

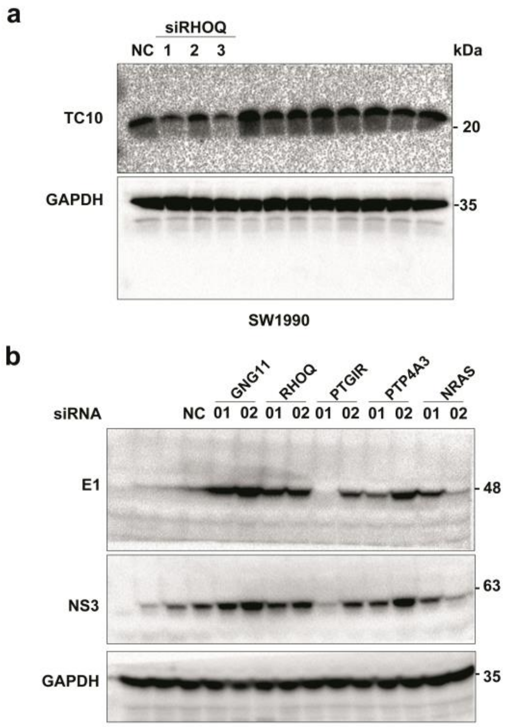

Supplementary Figure 15. Full western blots. Blots for Supplementary figure 6 (a) and 7 (b).

Gene Symbol		Gene Symbol		Gene Symbol		Gene Symbol		Gene Symbol	
1	MTMR7	44	INPP5E	87	BCHE	130	ACACB	173	WWTR1
2	PNPLA3	45	MBOAT2	88	PIP5K1B	131	ABCB4	174	EGFL8
3	TPTE2P6	46	HADHB	89	LPCAT4	132	ESRRA	175	MED27
4	PIK3C3	47	SLC44A2	90	ABHD4	133	ELOVL1	176	NCOR2
5	PLA2G3	48	MTMR1	91	PLB1	134	PPARGC1A	177	MCEE
6	SLC44A5	49	DDHD1	92	PGS1	135	ACSL6	178	ELOVL2
7	LPCAT2	50	ETNK1	93	MTMR2	136	ACADS	179	SLC25A20
8	CHPT1	51	PIP4K2C	94	PIP5K1A	137	MED31	180	MED9
9	PIKFYVE	52	CHKB	95	ETNK2	138	PEX11A	181	ELOVL3
10	FIG4	53	PIK3CD	96	PIK3C2A	139	SMARCD3	182	GPD1
11	INPP4A	54	PLA2G4A	97	PLA2G12A	140	ACOT7	183	MED15
12	PCYT1B	55	PIP5K1C	98	PTDSS1	141	MED25	184	HSD17B12
13	LPGAT1	56	SACM1L	99	INPPL1	142	PRKAA2	185	LPIN1
14	PLA2G2A	57	DDHD2	100	ACHE	143	PPARG	186	MED8
15	PI4KB	58	PITPNB	101	ARF3	144	PTPLA	187	BDH1
16	LPIN1	59	CHKA	102	PIK3R3	145	NRF1	188	ACADL
17	LPIN3	60	AGPAT9	103	ARF1	146	ELOVL5	189	CYP4A11
18	CDS2	61	LCLAT1	104	PISD	147	AGPAT2	190	TECRL
19	EPT1	62	PNPLA2	105	PLA2G4C	148	PRKAB2	191	ACLY
20	PLA2G6	63	GNPAT	106	PTEN	149	THEM4	192	ACADVL
21	PI4K2A	64	GPAT2	107	PTDSS2	150	RORA	193	ANGPTL4
22	PIK3CA	65	GPD1L	108	MTMR14	151	LPIN3	194	ELOVL4
23	PIK3C2B	66	MTMR4	109	PLA2G16	152	ME1	195	NCOA1
24	PIK3CB	67	DGAT2	110	CRLS1	153	GPD1L	196	PPARGC1B
25	MTMR3	68	AGPAT1	111	PNPLA8	154	ALAS1	197	NCOR1
26	CEPT1	69	DGAT1	112	AGPAT5	155	HMGCL	198	NCOA3
27	PLD3	70	AGPAT2	113	MTM1	156	NR1H4	199	TBL1X
28	ABHD3	71	GPCPD1	114	PLBD1	157	ACOX1	200	PTPLAD2
29	TAZ	72	INPP5K	115	PLD6	158	CPT1B	201	SULT2A1
30	MGLL	73	VAC14	116	PIK3CG	159	HMGCS2	202	ACAT1
31	JMJD7	74	CPNE1	117	SLC44A1	160	SCD	203	ACOT8
32	JMJD7	75	AGPAT4	118	OCRL	161	NFYC	204	PCCB
33	PLD4	76	MTMR6	119	PLA2G1B	162	DGAT2	205	HSD17B3
34	CDS1	77	CHAT	120	GPD1	163	DGAT1	206	CD36
35	INPP5D	78	PIK3R5	121	PIK3R1	164	TXNRD1	207	THEM5
36	PIP4K2B	79	CPNE6	122	PTPMT1	165	GK	208	SREBF2
37	HADHA	80	INPP5J	123	MBOAT7	166	ACSBG1	209	CYP1A1
38	AGPAT6	81	PLA2G4F	124	MED1	167	MED23	210	ACOT4
39	PCYT1A	82	PLA2G2E	125	FASN	168	ACOT9	211	AGK
40	PI4K2B	83	PLD2	126	PTPLAD1	169	TEAD2		
41	CPNE3	84	PEMT	127	ACOT13	170	HMGCLL1		
42	PIK3R6	85	GPAM	128	MED21	171	NR1D1		
43	MBOAT1	86	SLC44A4	129	SLC25A1	172	FABP1		

Supplementary Table 1. Fatty acid metabolism-related genes in Reactome database.

	Gene Symbol		Gene Symbol		Gene Symbol		Gene Symbol
1	MTMR7	42	PIK3R6	83	PLD2	124	PLD1
2	PNPLA3	43	MBOAT1	84	PEMT	125	PIP4K2A
3	TPTE2P6	44	INPP5E	85	GPAM	126	PLA2G2F
4	PIK3C3	45	MBOAT2	86	SLC44A4	127	CDIPT
5	PLA2G3	46	HADHB	87	BCHE	128	AGPAT3
6	SLC44A5	47	SLC44A2	88	PIP5K1B	129	LPCAT3
7	LPCAT2	48	MTMR1	89	LPCAT4	130	SYNJ1
8	CHPT1	49	DDHD1	90	ABHD4	131	CPNE7
9	PIKFYVE	50	ETNK1	91	PLB1	132	PLA2G2D
10	FIG4	51	PIP4K2C	92	PGS1	133	PCYT2
11	INPP4A	52	CHKB	93	MTMR2	134	PLA2G4D
12	PCYT1B	53	PIK3CD	94	PIP5K1A	135	PIK3R4
13	LPGAT1	54	PLA2G4A	95	ETNK2	136	SLC44A3
14	PLA2G2A	55	PIP5K1C	96	PIK3C2A	137	SYNJ2
15	PI4KB	56	SACM1L	97	PLA2G12A	138	PHOSPHO1
16	LPIN1	57	DDHD2	98	PTDSS1	139	TPTE
17	LPIN3	58	PITPNB	99	INPPL1	140	LPCAT1
18	CDS2	59	CHKA	100	ACHE	141	LPIN2
19	EPT1	60	AGPAT9	101	ARF3	142	ETNPPL
20	PLA2G6	61	LCLAT1	102	PIK3R3	143	PLA2G10
21	PI4K2A	62	PNPLA2	103	ARF1	144	PLA2G5
22	PIK3CA	63	GNPAT	104	PISD	145	PIK3C2G
23	PIK3C2B	64	GPAT2	105	PLA2G4C	146	INPP4B
24	PIK3CB	65	GPD1L	106	PTEN		
25	MTMR3	66	MTMR4	107	PTDSS2		
26	CEPT1	67	DGAT2	108	MTMR14		
27	PLD3	68	AGPAT1	109	PLA2G16		
28	ABHD3	69	DGAT1	110	CRLS1		
29	TAZ	70	AGPAT2	111	PNPLA8		
30	MGLL	71	GPCPD1	112	AGPAT5		
31	JMJD7	72	INPP5K	113	MTM1		
32	JMJD7	73	VAC14	114	PLBD1		
33	PLD4	74	CPNE1	115	PLD6		
34	CDS1	75	AGPAT4	116	PIK3CG		
35	INPP5D	76	MTMR6	117	SLC44A1		
36	PIP4K2B	77	CHAT	118	OCRL		
37	HADHA	78	PIK3R5	119	PLA2G1B		
38	AGPAT6	79	CPNE6	120	GPD1		
39	PCYT1A	80	INPP5J	121	PIK3R1		
40	PI4K2B	81	PLA2G4F	122	PTPMT1		
41	CPNE3	82	PLA2G2E	123	MBOAT7		

Supplementary Table 2. Phospholipid metabolism-related genes in Reactome database.

Gene Symbol		Gene Symbol	
1	ALDH3B1	41	SGMS2
2	ARSD	42	CTSA
3	SPTLC3	43	DEGS1
4	ASAH1	44	CPTP
5	PRKD1	45	GALC
6	DEGS2	46	ARSG
7	OSBP	47	CERS2
8	ENPP7	48	GLB1L
9	GM2A	49	GLB1L
10	PPAP2A	50	SPTLC1
11	NEU1	51	ACER1
12	ALDH3A2	52	SMPD2
13	ARSJ	53	SMPD3
14	SUMF1	54	SPHK1
15	SPTLC2	55	NEU2
16	SGPP1	56	ACER3
17	ARSF	57	CSNK1G2
18	ARSK	58	GLA
19	ARSE	59	SMPD4
20	ARSA	60	GBA2
21	UGT8	61	UGCG
22	PSAP	62	ARSI
23	PPAP2C	63	PPM1L
24	HEXB	64	CERS4
25	HEXA	65	CERS5
26	CERS1	66	SGPP2
27	SUMF2	67	ARSB
28	GBA	68	FA2H
29	CERS6	69	KDSR
30	SPHK2		
31	CERS3		
32	PPAP2B		
33	NEU3		
34	VAPA		
35	SGPL1		
36	SMPD1		
37	VAPB		
38	CERK		
39	GLTP		
40	STS		

Supplementary Table 3. Sphingolipid metabolism-related genes in Reactome database.

	Gene Symbol
1	HMGCS1
2	HMGCR
3	MVK
4	PMVK
5	MVD
6	FDPS
7	GGPS1
8	FDFT1
9	SQLE
10	LSS
11	CYP51A1
12	TM7SF2
13	MSMO1
14	NSDHL
15	HSD17B7
16	DHCR24
17	EBP
18	SC5D
19	DHCR7
20	IDI1
21	IDI2
22	LBR
23	ARV1

Supplementary Table 4. Cholesterol biosynthesis-related genes in Reactome database.

	Gene Symbol		Gene Symbol
1	ALDH3B1	35	PPP1R16B
2	BROX	36	PRICKLE1
3	CENPE	37	PRICKLE2
4	CLN3	38	RAB28
5	CNP	39	RAP2A
6	CPLX3	40	RASD1
7	CPLX4	41	ERAS
8	FAM127A	42	NRAS
9	DNAJA1	43	KRAS
10	DNAJA4	44	HRAS
11	DNAJA2	45	RHEBL1
12	GNG13	46	RHEB
13	GNGT1	47	RASD2
14	GNG11	48	RHOQ
15	GNGT2	49	RHOB
16	GBP1	50	RHOJ
17	INPP5B	51	GRK1
18	INPP5A	52	RND3
19	ICMT	53	RRAS2
20	PHKA2	54	RASL10A
21	PHKA1	55	STK11
22	PHKB	56	PTP4A1
23	LMNB1	57	PTP4A3
24	LMNA	58	PTP4A2
25	LMNB2	59	USP32
26	NAP1L1	60	UCHL1
27	PLA2G4C	61	YKT6
28	PALM3		
29	PALM		
30	PALM2		
31	PDE6A PDEA		
32	PEX19		
33	PTGIR		
34	PPP1R16A		

Supplementary Table 5. FT-regulating candidates in Swiss-Prot database.

Gene Symbol	Sense/Antisense	Sequence
HMGCS1	sense	CATTAGACCGCTGCTATTCTGTC
	antisense	TTCAGCAACATCCGAGCTAGA
HMGCR	sense	TGATTGACCTTTCCAGAGCAAG
	antisense	CTAAAATTGCCATTCCACGAGC
MVK	sense	GGAGCAAGGTGATGTCACAAC
	antisense	CGGCAGATGGACAGGTATAAGT
MVD	sense	GGACCGGATTTGGCTGAATG
	antisense	CCCATCCCGTGAGTTCCTC
FDPS	sense	TGTGACCGGCAAAATTGGC
	antisense	GCCCGTTGCAGACACTGAA
M1 NS1	sense	GTTCCAACAGGCGTCACCATC
	antisense	ACACATTCTTGTCTAGCACAGTCC
IRF9	sense	(CAGCAGGAACCCTCCCTAAC
	antisense	GAAAGGCCACACACCTGAGTT
DDX58	sense	ATCCCAGTGTATGAACAGCAG
	antisense	GCCTGTAACTCTATACCCATGTC
MDA5	sense	TCACAAGTTGATGGTCCTCAAGT
	antisense	CTGATGAGTTATTCTCCATGCCC
IFNB	sense	GCTTGGATTCCTACAAAGAAGCA
	antisense	ATAGATGGTCAATGCGGCGTC
IRF3	sense	AGAGGCTCGTGATGGTCAAG
	antisense	AGGTCCACAGTATTCTCCAGG
IRF7	sense	CCCACGCTATACCATCTACCT
	antisense	GATGTCGTCATAGAGGCTGTTG
TBP1	sense	GAGCTGTGATGTGAAGTTTCC
	antisense	TCTGGGTTTGATCATTCTGTAG
ACTB	sense	GATCATTGCTCCTCCTGAGC
	antisense	ACTCCTGCTTGCTGATCCAC

Supplementary Table 6. The amplification primers.
